# Increased B-Type Natriuretic Peptide Concentration Is Associated with Reduced Coronary Vasoreactivity in Patients with Dilated Cardiomyopathy but Not in Healthy Young Subjects

**DOI:** 10.5402/2011/638764

**Published:** 2011-04-11

**Authors:** Jan Sundell, Erik Engblom, Juhani Koistinen, Antti Ylitalo, Hanna Laine, Riikka Kalliokoski, K. E. Juhani Airaksinen, Jeroen J. Bax, Juhani Knuuti

**Affiliations:** ^1^Turku PET Centre, University of Turku, P.O. Box 52, 20521 Turku, Finland; ^2^Department of Medicine, University of Turku, 20520 Turku, Finland; ^3^Department of Medicine, Satakunta Central Hospital, 28500 Pori, Finland; ^4^Department of Cardiology, Leiden University, 2333 ZA Leiden, The Netherlands

## Abstract

*Background/Aims*. Natriuretic peptides are associated with the cardiovascular disease risk under a range of different circumstances. However, less is known about whether this association is found also in young healthy subjects. *Methods*. 9 patients with dilated cardiomyopathy and 26 healthy young subjects were studied. The myocardial blood flow measurements were performed basally and during adenosine infusion using PET. *Results*. S-proBNP concentrations were significantly higher (2153 ± 1964 versus 28 ± 17 ng/L, *P* = .000002) and adenosine-stimulated flow lower (1.6 ± 0.8 versus 3.6 ± 1.1 mL·g^−1^·min^−1^, *P* = .00001) in patients with dilated cardiomyopathy when compared to healthy subjects. S-proBNP concentration was inversely associated with adenosine stimulated flow in patients with dilated cardiomyopathy (*r* = −0.75, *P* = .019) but not in healthy subjects (*r* = −0.06, *P* = .84). *Conclusions*. Natriuretic peptides are inversely associated with coronary vasoreactivity in patients with dilated cardiomyopathy but not in healthy young subjects. Since reduced coronary vasoreactivity seems to be one of the earliest abnormalities in the development of coronary artery disease, this might indicate that natriuretic peptides are not predictor of cardiovascular disease risk in healthy young subjects.

## 1. Introduction

Natriuretic peptides are released in response to increased ventricular wall stress [1] and myocardial ischemia [2]. Serum natriuretic peptide concentrations have been demonstrated to correlate with systolic dysfunction, and NYHA class and also have prognostic value [3]. Recently, it has been found that these peptides may also be indicators of cardiovascular events even in healthy, elderly individuals [4]. However, less is known whether this association is found also in healthy young subjects.

Reduced coronary vasoreactivity seems to be one of the earliest abnormalities in the development of coronary artery disease. Although high costs and limited availability prevent the wider use of the method, PET enables noninvasive, quantitative, and accurate measurements of myocardial blood flow in humans [5]. Using PET, coronary vasoreactivity has been found to be reduced in patients with coronary artery disease, diabetes, hypertension, and dyslipidemias, and it appears to correlate also with the risk factors of coronary artery disease even in healthy young subjects [6]. 

The present PET study was designed to evaluate whether natriuretic peptides are associated with coronary vasoreactivity in patients with dilated cardiomyopathy and in healthy young subjects. Myocardial blood flow was determined basally and during adenosine infusion using PET and oxygen-15-labelled water ([^15^O]H_2_O).

## 2. Methods

### 2.1. Subjects

The characteristics of the patients with dilated cardiomyopathy are summarized in Table 1. Nine subjects with symptomatic heart failure, idiopathic dilated cardiomyopathy (LV ejection fraction <45%, left bundle branch block), and cardiac resynchronization therapy (CRT) were included. No patient had atrial fibrillation or atrioventricular conduction disorders. All patients were clinically stable, treated with CRT and optimized medical therapy, and did not have evidence of decompensated heart failure at the time of the study. Moreover, to study the association between natriuretic peptides and coronary vasoreactivity in healthy subjects, twenty-six asymptomatic nonsmoking men (age 34 ± 6 years, BMI 26 ± 2 kg/m^2^, LV ejection fraction 67 ± 5%) were investigated. These subjects were healthy as judged by history and physical examination and were not taking any medication. All healthy subjects were normotensive and had normal glucose tolerance and lipid profile. All of the electrocardiograms, stress echocardiograms, and echocardiographically determined LV masses, dimensions, and functions were normal in studied healthy subjects. The study was conducted according to the guidelines of the Declaration of Helsinki, and the Ethics Committee of the Turku University Central Hospital accepted the study protocol. Each subject gave written informed consent.

### 2.2. Study Design

The study was performed at the Turku PET Centre (Turku University Central Hospital, Turku, Finland). In patients with dilated cardiomyopathy, the dose of ß-blockade was gradually reduced during 6 days before the PET studies (to avoid rebound effect), withdrawn completely 2 days before the PET studies, and restarted again after the PET studies. Other cardiac medication was continued during the PET studies. Pacemaker was switched off for 24 hours before the PET studies. In all subjects, PET studies were performed after an overnight fast. Additionally, the subjects were instructed to avoid all caffeine-containing drinks and foods for 12 hours before the PET studies. 

Serum N-Terminal probrain natriuretic peptide (S-proBNP) was determined immediately preceding the PET studies. Myocardial perfusion was measured using ^15^O-water and PET at rest and then after administration of adenosine. The subjects' electrocardiogram and heart rate were monitored continuously during the studies. Blood pressure was monitored throughout the procedures with an automatic oscillometric blood pressure monitor (OMRON HEM-705C, Omron Healthcare, Hamburg, Germany) during all PET studies.

### 2.3. Measurement of Myocardial Perfusion

The positron emitting tracer, [^15^O]water, was produced as previously described [7]. The subjects were positioned supine in a 15-slice ECAT 931/08-12 tomograph (Siemens/CTI Inc., Knoxville, TN, USA). After the transmission scan, myocardial perfusion was measured with an intravenous injection of [^15^O] water (~1.5 GBq) at rest and 60 seconds after intravenous administration of adenosine (140 *μ*g/kg per minute). Dynamic scan lasted for 6 minutes (6 × 5, 6 × 15, and 8 × 30 seconds). All data were corrected for dead time, radioactive decay, and photon attenuation. Images were processed using an iterative median root prior reconstruction algorithm [8].

### 2.4. Analysis and Calculation of Myocardial Blood Flow

Regions of interest (ROI) were drawn in the lateral, anterior and septal wall of the left ventricle in four representative transaxial slices in each study as previously described [7]. The ROI outlined in the baseline images was copied to the images obtained after adenosine administration. Values of myocardial blood flow (expressed in mL/gram of tissue per minute) were calculated according to the previously published method using the single-compartment model [9]. The arterial input function was obtained from the LV time activity curve using a previously validated method [10], in which corrections were made for the limited recovery of the LV ROI and the spillover from the myocardial signals. The average blood flow of the lateral and anterior part of the myocardium showed the lowest coefficient of variation and was used in further analysis. Coronary flow reserve was defined as the ratio of the myocardial blood flow during adenosine infusion to flow at baseline.

### 2.5. Assessment of Natriuretic Peptide

S-proBNP was measured using an electrochemiluminometric (ECLIA) method (reagent kit: Roche, Mannheim, Germany; instrument: Elecsys, Roche, Mannheim, Germany; analysed at Limbach Laboratory, Germany).

### 2.6. Statistical Analysis

The results are expressed as mean ± standard deviation (SD). Student's paired *t*-test was used within the group comparisons and Student's unpaired *t*-test when two different groups were compared. For correlation analysis, Spearman's correlation coefficients were calculated. *P* values less than  .05 were interpreted as statistically significant. All statistical tests were performed with SAS statistical analysis system (SAS Institute Inc., Gary, N.C., USA).

## 3. Results

### 3.1. Hemodynamic Measurements during PET

Heart rates, blood pressures, and rate-pressure products during the PET study are presented in Table 2. Adenosine infusion induced a significant increase in heart rate and rate-pressure products.

### 3.2. Myocardial Blood Flow Values

Basal myocardial blood flow was not different between patients with dilated cardiomyopathy (0.78 ± 0.33 mL·g^−1^·min^−1^) and healthy subjects (0.81 ± 0.21 mL·g^−1^·min^−1^, NS). Adenosine stimulated flow was lower in patients with dilated cardiomyopathy when compared to healthy subjects (1.6 ± 0.8 versus 3.6 ± 1.1 mL·g^−1^·min^−1^, *P* = .00001). Concordantly, coronary flow reserve was lower in patients with dilated cardiomyopathy when compared to healthy subjects (2.1 ± 0.7 versus 3.6 ± 1.1, *P* = .000004).

### 3.3. Laboratory Parameters

**Figure 1:**
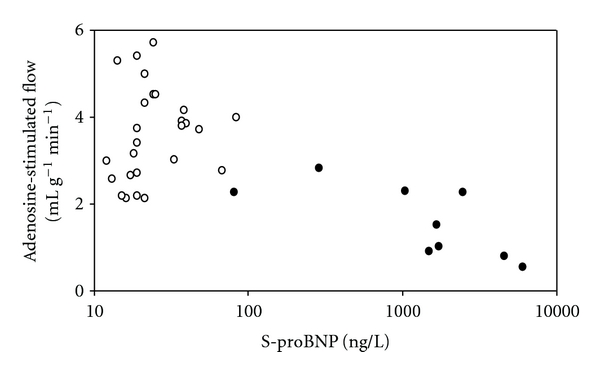
Serum proBNP concentration (logarithm scale) was inversely associated with adenosine stimulated flow in patients with dilated cardiomyopathy (black circle: *r* = −0.75, *P* = .019) but not in healthy subjects (open circle: *r* = −0.06, *P* = .84).

Serum proBNP concentrations were significantly higher in patients with dilated cardiomyopathy when compared to healthy subjects (2153 ± 1964 versus 28 ± 17 ng/L, *P* = .000002). LV ejection fraction was inversely (*r* = −0.69, *P* = .037) and NYHA class positively correlated (*r* = 0.82, *P* = .0066) with serum proBNP concentration in patients with dilated cardiomyopathy. Serum proBNP concentration was not significantly associated with the basal myocardial blood flow in patients with dilated cardiomyopathy and healthy subjects (NS). Serum proBNP concentration was inversely associated with adenosine stimulated flow in patients with dilated cardiomyopathy (*r* = −0.75, *P* = .019) but not in healthy subjects (*r* = −0.06, *P* = .84) (Figure 1). Concordantly, serum proBNP concentration was inversely associated with coronary flow reserve in patients with dilated cardiomyopathy (*r* = −0.73, *P* = .025) but not in healthy subjects (NS).

## 4. Discussion

As expected, in the present study patients with dilated cardiomyopathy and heart failure had increased serum natriuretic peptide concentrations and reduced adenosine stimulated myocardial blood flow when compared to healthy young subjects. However, we demonstrated that natriuretic peptide concentrations were inversely associated with adenosine stimulated flow in patients with dilated cardiomyopathy but not in healthy young subjects. 

Patients with dilated cardiomyopathy have reduced coronary vasoreactivity in the absence of ischemic heart disease [11, 12]. The exact mechanism of reduced coronary vasoreactivity in these patients is still unknown, but it may relate to coronary endothelial and microcirculatory dysfunction [13]. In the present study, the myocardial blood flow values in patients with dilated cardiomyopathy, and healthy subjects agree with values reported by other cardiovascular studies [11, 12]. As expected, patients with dilated cardiomyopathy had reduced adenosine stimulated myocardial blood flow and coronary flow reserve when compared to healthy subjects. 

Natriuretic peptides are released in response to increased ventricular wall stress [1]. Serum natriuretic peptide concentrations have been demonstrated to correlate with systolic dysfunction, NYHA class and also have prognostic value [3]. These associations between LV ejection fraction, symptoms, and natriuretic peptides were also found in the present study. Recently, a strong inverse correlation has been found between serum proBNP concentration and coronary blood flow reserve by using Doppler ultrasound techniques [14]. In the present study, only a limited number of patients were investigated. However, the study protocol was complicated and laborious. Despite the limited study population, we were able to demonstrate that natriuretic peptides are inversely associated with coronary vasoreactivity in patients with dilated cardiomyopathy. This indicate that vascular dysfunction parallels with ventricular dysfunction. 

In addition to heart failure serum, natriuretic peptides are substantially increased during acute coronary syndromes [15]. Thus, myocardial ischemia appears to be also a key stimulus for these peptides. Moreover, it has been speculated that natriuretic peptides might be even early indicators of cardiovascular impairment [4]. Since coronary dysfunction seems to be one of the earliest abnormalities in the development of coronary artery disease [16], increased concentrations of natriuretic peptides may predict future coronary artery disease in patients with dilated cardiomyopathy. This would indicate that especially patients with dilated cardiomyopathy and increased natriuretic peptide concentration need intensive treatment of cardiovascular risk factors.

Recently, it has been found that natriuretic peptides may be indicators of cardiovascular events even in healthy elderly adults [4]. In the present study, healthy subjects were not matched with the patients, since we wanted to specifically investigate the association between natriuretic peptides and coronary vasoreactivity in healthy young men who had no risk factors such as obesity, smoking, diabetes, and dyslipidemia, which are known to further reduce coronary vascular reactivity [6]. We did not find any correlation between serum proBNP concentration and coronary vasoreactivity in these subjects. This might indicate that natriuretic peptides are not predictor of cardiovascular disease risk in healthy young subjects.

## 5. Summary

The present study demonstrates that natriuretic peptides are inversely associated with coronary vasoreactivity in patients with dilated cardiomyopathy indicating that vascular dysfunction parallels with ventricular dysfunction. However, we did not find any correlation between serum proBNP concentration and coronary vasoreactivity in healthy young subjects. This might indicate that natriuretic peptides are not predictor of cardiovascular disease risk in healthy young subjects.

##  Conflict of Interests

The authors declared that there is no conflict of interests. 

## Figures and Tables

**Table 1:** Characteristics of the patients with dilated cardiomyopathy [mean (SD)].

Gender (M : F)	7 : 2
Age (years)	58 (12)
Body mass index (kg/m^2^)	31.7 (5.0)
Blood pressure (mmHg)	114/73 (13/6)
NYHA class	2.3 (0.5)
LV ejection fraction (%)	32.0 (8.3)
LV mass (g)	315.5 (102.7)
Mitral regurgitation grade	2.0 (0.6)
QRS duration (ms)	138 (14)
Medications:	
Diuretics	9/9
ACE-inhibitors	5/9
Angiotensin II blocker	4/9
ß-blockers	9/9
Digoxin	1/9

**Table 2:** Hemodynamic data during PET studies [mean (SD)].

	Basal	Adenosine
Heart rate (beats/min)		
Patients with DCM	72 (12)	83 (14)*
Healthy subjects	60 (9)	98 (10)*
Systolic bp (mmHg)		
Patients with DCM	115 (16)	116 (11)
Healthy subjects	122 (12)	124 (14)
Diastolic bp (mmHg)		
Patients with DCM	71 (10)	73 (7)
Healthy subjects	71 (11)	70 (11)
RPP (mmHg/min)		
Patients with DCM	8193 (1245)	9520 (1692)*
Healthy subjects	7064 (1092)	12083 (1719)*

DCM: dilated cardiomyopathy, bp: blood pressure, RPP: rate pressure product (systolic bp × heart rate).

*P < .05 versus basal.

## Abbreviations

CRT: Cardiac resynchronization therapy
DCM: Dilated cardiomyopathy
LV: Left ventricular
PET: Positron emission tomography
NYHA: New York Heart Association class
S-proBNP: Serum N-Terminal probrain natriuretic peptide
[^15^O]water: Oxygen-15-labeled water
ROI: Region of interest.
